# Neuropathic Itch: Routes to Clinical Diagnosis

**DOI:** 10.3389/fmed.2021.641746

**Published:** 2021-02-24

**Authors:** Manuel Pedro Pereira, Henning Wiegmann, Konstantin Agelopoulos, Sonja Ständer

**Affiliations:** Department of Dermatology and Center for Chronic Pruritus, University Hospital Münster, Münster, Germany

**Keywords:** neuropathic itch, chronic itch, dysesthesia, pain, small-fiber neuropathy, diagnostic work-up, intraepidermal nerve fiber density, magnet resonance imaging

## Abstract

Neuropathic itch occurs due to damage of neurons of the peripheral or central nervous system. Several entities, including metabolic, neurodegenerative, orthopedic, infectious, autoimmune, malignant, and iatrogenic conditions, may affect the somatosensory system and induce neuropathic itch. Due to the complex nature of neuropathic itch, particularly concerning its clinical presentation and possible etiological factors, diagnostic work-up of this condition is challenging. A detailed medical history, especially in regard to the itch, and a comprehensive physical examination are relevant to detect characteristic signs and symptoms of neuropathic itch and to rule out other possible causes for chronic itch. Complementary diagnostic exams, especially laboratory tests, determination of the intraepidermal nerve fiber density via a skin biopsy and radiological examinations may be indicated to confirm the diagnosis of neuropathic itch and to identify underlying etiological factors. Functional assessments such as quantitative sensory testing, nerve conduction studies, evoked potentials, or microneurography may be considered in particular cases. This review article provides a comprehensive overview of the diagnostic work-up recommended for patients with neuropathic itch.

## Introduction

The International Forum for the Study of Itch (IFSI) defines neuropathic itch as itch occurring due to an injury of neurons of the peripheral or central nervous system (1). It is estimated that 8% of chronic itch cases have a neuropathic origin (2). However, epidemiological studies investigating the prevalence and incidence of neuropathic itch are still missing.

Damage at any site of the somatosensory system, including peripherally nerve fibers, nerve plexuses and ganglia, and centrally the spinal cord, brainstem, thalamus or cortex, may lead to neuropathic itch (3).

Several conditions affecting the peripheral nervous system are associated with neuropathic itch (Table 1). In small-fiber neuropathy (SFN), which results from injured C- and Aδ fibers (4), itch and pain may occur localized (mostly distally at the feet) or generalized. It may result from a myriad of conditions such as metabolic (e.g., diabetes), infectious, autoimmune and genetic diseases. Also drugs (e.g., chemotherapy) and alcohol may induce SFN (5, 6). Scars and burn-injuries are often associated with itch, likely due to damage of cutaneous nerves (7, 8). Postherpetic neuralgia is a frequent cause of neuropathic itch at the site of the affected peripheral nerve (9). Compression or irritation of different neural structures may induce localized neuropathic itch along the corresponding dermatome, as is the case in brachioradial pruritus (radiculopathy at C3–C6), notalgia paresthetica (dorsal rami of posterior nerves at T2–T6), cheiralgia paresthetica (radial nerve), meralgia paresthetica (lateral femoral cutaneous nerve), and gonyalgia paresthetica (infrapatellar branch of the saphenous nerve) (2, 10–13). Genitoanal itch, a common pruritic condition, can also have a neuropathic origin, due to a lumbosacral radiculopathy. Injury of the trigeminal nerve may cause facial itch leading often to manipulation of the skin with ulceration, a condition termed trigeminal trophic syndrome (14).

**Table 1:** Neuropathic pruritic conditions.

**Disease**	**Etiology**	**Clinical features**	**Main work-up**
**Peripheral nervous system**			
Small fiber neuropathy	Metabolic, drug-induced, infectious, or genetic origin	Itch starts usually distally and may generalize	IENFD, QST
Scars and burns	Iatrogenic or traumatic	Itch on lesional tissue	Clinical diagnosis
Radiculopathies	Compression of a peripheral nerve by degenerative alterations or space-occupying lesions	Itch and dysesthesias at the affected dermatome	MRI or CT scan, IENFD
Postherpetic neuralgia	Damage of peripheral nerve by the varicella zoster virus	Itch and dysesthesias at the affected dermatome	Clinical diagnosis
Trigeminal trophic syndrome	Injury of the sensory fibers of the trigeminal nerve	Unilateral dysesthesia and hypoesthesia of the central face. Self-induced ulceration of the nasal ala, cheek, and upper lip	Clinical diagnosis, MRI
**Central nervous system**			
Space occupying lesions	Tumors, abscesses, vascular lesions, syringomyelia	Clinical features according to affected neural structures	Neuroimaging (MRI/CT scan)
Stroke	Ischemic or hemorrhagic	Generalized or unilateral itch	Neuroimaging (CT scan)
Multiple sclerosis	Demyelinating disease	Generalized itch or localized at the head and upper back	MRI, analysis of cerebrospinal fluid (IgG oligoclonal bands), evoked potentials
Neuromyelitis optica	Demyelinating disease	Depending on injured spinal level	MRI, autoantibodies against aquaporin 4
Infectious diseases	Meningitis, encephalitis, prion disease	Depending on damaged neural structures	Neuroimaging (MRI/CT scan), analysis of cerebrospinal fluid, blood tests
Traumatic brain or spinal cord lesions	Accidents or iatrogenic lesions	Depending on damaged neural structures	Neuroimaging (MRI/CT scan)

*Etiological factors, clinical features and diagnostic work-up of neuropathic pruritic conditions are shown. CT, computed tomography; IENFD, intraepidermal nerve fiber density; QST, quantitative sensory testing; MRI, magnet resonance imaging*.

At central level, space-occupying lesions such as abscesses, cysts, tumors, vascular malformations or syringomyelia may originate neuropathic itch (15, 16). Also neural damage induced by trauma or meningitis has been associated with the occurrence of itch, while unilateral itch has been reported after a stroke (17, 18). Additionally, itch has been reported in neuroinflammatory conditions as for instance multiple sclerosis or neuromyelitis optica (19, 20).

Neuropathic itch should be differentiated from other etiologies possibly underlying itch. Table 2 provides an overview of potential etiologies of chronic itch as defined by the IFSI, including clinical examples and distinctive clinical features.

**Table 2:** Etiological classification of itch.

**Etiology**	**Clinical examples**	**Clinical features**
Dermatological	Atopic dermatitis, cutaneous T-cell lymphoma, psoriasis, urticaria	Primary skin alterations (e.g., eczema) can be found upon inspection
Systemic	Cholestasis, diabetes, drug-induced, myeloproliferative disorders, renal insufficiency, solid tumors	Temporal correlation between onset of systemic disease and begin of itch may hint toward a systemic origin of itch Onset of itch at the trunk is typical for diabetes, while begin of itch at the palms and soles suggests a liver disease as an underlying cause
Neuropathic	Brachioradial pruritus, notalgia paresthetica, postherpetic neuralgia, small-fiber neuropathy	Itch usually starts localized according to the affected nerves. Symptomatic alleviation with application of cold or ice
Psychosomatic/psychiatric	Delusional parasitosis, somatoform pruritus	Onset of itch may occur after a significant life event (e.g., death of the partner, job loss)
Mixed	If more than one etiology is found	Occurs more frequently in older or multimorbid patients
Unknown	If no underlying etiology is found after diagnostic work-up	Diagnostic work-up should be repeated yearly if the cause for chronic itch remains unknown

*Adapted from Ständer et al. (1)*.

Owing to the multidimensional nature of neuropathic itch, particularly regarding its clinical presentation and possible underlying causes, substantial diagnostic efforts are necessary in the management of these patients. This review article focuses on the diagnostic work-up of patients with neuropathic itch, including the medical history and physical examination (Table 3), use of standardized questionnaires, laboratory tests, skin biopsies for the assessment of neurocutaneous alterations, radiological examinations, and functional tests (Table 4).

**Table 3:** Medical history and physical examination: relevant aspects for neuropathic itch.

	**Parameter**	**Comment**
**Medical history**		
Itch specific history	Intensity	Itch intensity informs about the disease severity and should be monitored during the course of the disease
	Sensory symptoms	Dysesthesias such as stinging, tingling or sensation like electroshocks are typical for neuropathic itch
	Daily pattern	Itch usually occurs in attacks in neuropathic itch
	Localization at onset	Localization of the itch at onset gives important hints regarding the affected site of the somatosensory system
	Localization in the course of disease	Generalization of itch beyond the initial localization at onset may occur, suggesting neuronal sensitization
	Alleviating factors	Alleviation of sensory symptoms with application of cool-packs or cold water is characteristic for neuropathic itch (ice-pack sign)
	Performed therapies	Neuropathic itch is expected to improve with gabapentinoids or opioid modulators, while antihistamines are usually ineffective
General medical history	Comorbidities	Assessment of comorbidities including atopic conditions is important to rule out non-neuropathic conditions leading to chronic pruritus. Additionally, systemic conditions (e.g., renal insufficiency) may limit therapeutic options
	Co-medication	Co-medication should be assessed to exclude possible drug interactions when planning an antipruritic therapy
**Physical examination**		
Dermatological examination	Dermatoses	Primary skin conditions as a possible cause for chronic itch should be ruled out by clinical examination (and eventually with skin biopsies). Importantly, dermatoses should be differentiated from secondary scratch lesions
	Scratch lesions	The distribution of scratch lesions (along with the sensory symptoms) may inform on the affected site of the somatosensory system. Additionally the amount of scratch lesions may serve as an indirect indicator of disease severity
Neurological examination	Alloknesis, Hyperknesis	Pruritic response to a non-pruritic stimulus (e.g., cotton swab) and augmented pruritic response to a pruritic stimulus (e.g., skin challenge with cowhage) suggest neuronal sensitization
	Mapping of dysesthesias	Mapping areas of dysesthesia (and alloknesis/hyperknesis) is helpful in localized pruritic neuropathic syndromes, as it may give hints on the affected neural structure

**Table 4:** Complementary diagnostic procedures in neuropathic itch.

**Diagnostic procedure**	**Comment**
Laboratory tests	• Exclusion of systemic disease as underlying cause for chronic pruritus • Exploratory work-up to investigate possible conditions inducing small-fiber neuropathy • Confirmatory tests of neurological disease (e.g., oligoclonal bands in multiple sclerosis) • Genetic work-up if hereditary conditions (e.g., genetic small-fiber neuropathy and channelopathy) is suspected
Skin biopsy	• Determination of the intraepidermal nerve fiber density to detect alterations of the cutaneous neuroanatomical architecture • Histology and/or direct immunofluorescence to rule out a primary skin condition
Radiological examinations	• Magnet resonance imaging (MRI)/computed tomography (CT) to identify space-occupying lesions affecting the central or peripheral nervous system • MRI (alternatively CT) to detect spinal disorders (e.g., degenerative alterations of the vertebral column, disc prolapse or herniation, compression of nerve roots or spinal nerves, neuroforaminal stenosis) in patients with pruritic compression syndromes • Diagnosis of neurological conditions potentially inducing itch (e.g., stroke, meningitis, multiple sclerosis)
Functional assessments	• Quantitative sensory testing to assess gain or loss of function of different populations of peripheral nerve fibers, including C and Aδ-fibers, and to detect possible signs of neuronal sensitization • Nerve conduction studies and electromyography to investigate impairment of large sensory (and motor) nerves • Microneurography and evoked potentials allow the assessment of selective nerve fibers, but are mostly used in research

## Medical History

**Figure 1:**
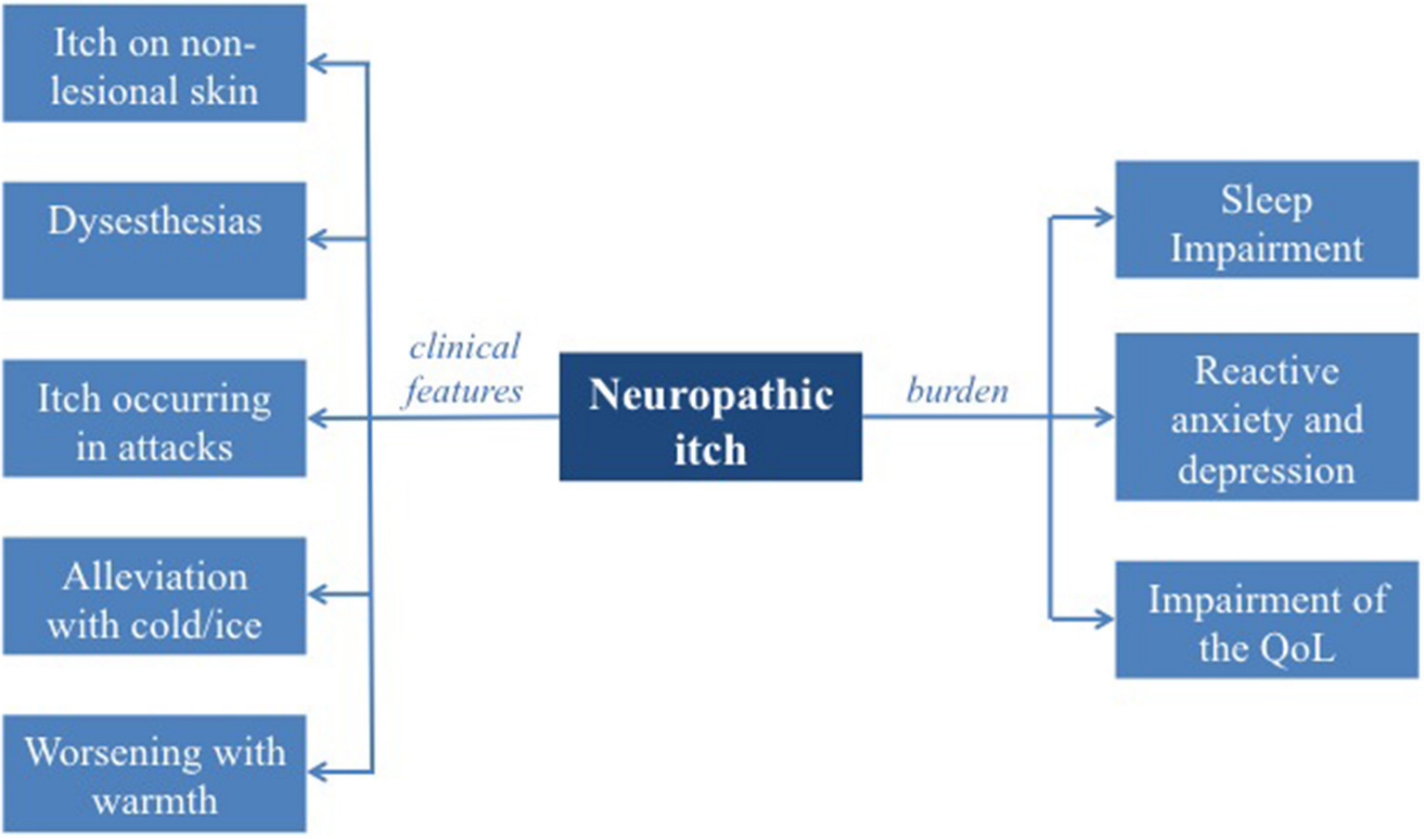
Neuropathic itch: clinical features and burden.

A detailed medical history is essential for the diagnosis of neuropathic itch and to exclude other possible etiologies for chronic pruritus. The itch characteristics should be asked in detail. The beginning of the pruritus and possible associated relevant events (e.g., herpes zoster prior to postherpetic neuralgia, or treatment with chemotherapy resulting in SFN) may give important clues of its etiology. Additionally, the appearance of the skin at the onset of the disease should be asked. Neuropathic itch starts on normal appearing skin, as no primary skin condition is present, but excoriations or chronic scratch lesions (e.g., chronic nodular prurigo or lichen simplex) may develop at a later stage due to ongoing scratching behavior (Figure 1) (21).

Also the localization of itch at the beginning and during the course of the disease should be addressed. The affected site of the somatosensory system is of paramount importance for the localization of itch. However, generalization of itch may occur after initial localized begin (22).

Pruritus intensity, accompanying sensory symptoms, fluctuation of the itch during the day and alleviating factors should be assessed. Typical for neuropathic itch is the presence of additional dysesthesias such as stinging and tingling, occurrence of itch in attacks and alleviation of the symptoms after application of cool-packs or cold water (Figure 1) (5, 23).

Moreover, information on previous antipruritic therapies and their effect and tolerance may help guiding the decision on further therapeutic proceedings (24, 25).

In addition to the itch specific history, the general medical history, especially regarding comorbidities and co-medication are important to rule other possible factors contributing to the development of pruritus and to inform on eventual limitations of therapeutic options due to drug interactions or systemic conditions as for instance impairment of renal or liver function (24, 25).

## Physical Examination

A comprehensive dermatological examination of the entire skin including mucosae is advised (24, 25). In neuropathic itch, no primary skin diseases are expected to be found. These should be differentiated from secondary lesions resulting from scratching such as excoriations or lichenification (24, 25). The distribution pattern of possible scratch marks and/or of the reported symptoms in localized pruritic conditions is essential for the correct diagnosis of the neuropathy. For instance, SFN manifests initially distally at the feet and advance proximally with the course of the disease, whereas neuropathic itch due to postherpetic neuralgia occurs at the affected dermatome. Brachioradial pruritus affects the outer aspects of the arms, while in notalgia paresthetica a circumscribed hyperpigmented area due to rubbing, mostly between the scapulae at the back, is characteristic (26). Unilateral itch should raise suspicion of a past stroke (17).

Alloknesis, i.e., induction of itch after application of a non-pruritic stimulus, and hyperknesis, i.e., augmented itch response to a pruritic stimulus, may occur in neuropathic itch (27) and should be assessed in these patients. A typical example for alloknesis often reported by patients is an intense itch perception after light touch of the skin in an affected site. These phenomena argue for neuronal sensitization processes, which contribute to the chronicity of the itch (28).

## Standardized Questionnaires

Standardized questionnaires, as for instance the *doleur neuropathique* (DN4) (29), PainDetect (30), or the Brief Pain Inventory (31), have been successfully developed to screen for neuropathic pain (32). For neuropathic itch no such tools were available until recently, when a score to differentiate neuropathic from non-neuropathic itch based on patient reported outcomes was proposed. Independent factors for neuropathic itch were the presence of twinges, absence of burning, worsening of the itch with activity, no worsening with stress and relief of itch with cold temperature. As a result, the Neuropathic Pruritus 5 (NP5) score was suggested based on the 5 independent factors for neuropathic itch. The presence of two out of five criteria yield a sensitivity of 76% and a specificity of 77% in discriminating neuropathic from non-neuropathic itch (33). For SFN a patient oriented survey including a question on itchy skin is in development (34).

## Skin Biopsy: Intraepidermal Nerve Fiber Density

Neurocutaneous morphological alterations are observed in neuropathic pruritic conditions. The determination of the intraepidermal nerve fiber density (IENFD) is the gold standard for the diagnosis of a SFN (5). Additionally, a reduction of the IENFD is also observed in neuropathic compression syndromes as for instance brachioradial pruritus (35). Thus, the examination of the epidermal neural architecture may provide important hints for the diagnosis of a neuropathic pruritic condition. Clinically, the magnitude of the decrease in IENFD seems to influence the perception of dysesthesias (5).

In order to determine the IENFD when neuropathic itch is suspected, a skin sample is obtained via a punch biopsy from non-lesional pruritic skin. Importantly, scratch lesions, scars or other skin conditions (e.g., eczema, skin infections) should be avoided when choosing the biopsy site, since such alterations may lead to false pathological findings. After staining of the skin sample with an axonal marker (e.g., protein gene product 9.5), nerve fibers crossing the basal membrane from the dermis into the epidermis are counted and divided by the length of the dermoepidermal junction. Fragments of nerve fibers in the epidermis and branching are not considered for the IENFD (36). Reference values are currently only available for the innervation site of the sural nerve (37). Therefore, this area (lateral lower leg) should be chosen for the biopsy, if patients report dysesthesias there. If another body site is affected, a skin sample from a non-affected symmetrical region should be obtained for comparison.

## Laboratory Tests

Laboratory tests are indicated to rule out non-neuropathic conditions potentially inducing chronic itch, as for instance renal retention parameters to exclude renal insufficiency, cholestasis parameters or complete blood count to screen for hemato-oncological diseases (24, 25). Moreover, disease specific tests should be performed in selected patients with suspicion of a neurologic condition (e.g., analysis of cerebrospinal fluid including histology if a brain tumor is suspected, or oligoclonal bands for the diagnosis of multiple sclerosis) (24).

Additionally, after a SFN is diagnosed laboratory investigations should be performed in order to identify possible causes. The assessment of glycosylated hemoglobin to rule out diabetes, vitamin B12 and folate serum levels, HIV and hepatitis B and C serology, TSH, and antinuclear antibodies constitute the most relevant assessments. Additionally, genetic tests may be considered for young patients with SFN of unclear origin to exclude a hereditary condition. However, in spite of a comprehensive work-up, the etiology of SFN remains unknown in a substantial number of cases (38).

## Radiological Examinations

Diagnostic imaging, especially magnet resonance imaging (MRI) and computed tomography (CT), is helpful to detect space-occupying lesions such as tumors, abscesses, vascular or inflammatory lesions and their anatomical relationship to peripheral or central neural structures. Medical imaging plays also a relevant role in the diagnosis of neurological conditions as e.g., stroke, meningitis or degenerative neuroinflammatory diseases, which potentially induce neuropathic itch.

MRI [alternatively CT, high-resolution sonography or MR neurography (39)] is oftentimes used in the diagnostic work-up of pruritic neuropathic compression syndromes to identify underlying pathologies such as compression of nerve roots or of the spinal cord, disc prolapse or herniation, degenerative vertebral alterations, osteophytes or neuroforaminal stenosis (10, 11, 26). While in brachioradial pruritus there is a clear correlation between MRI findings and the localization of dysesthesias, such a relationship is not so clear for notalgia paresthetica (40).

## Functional Assessments

Morphological investigations of neuroanatomical alterations may be complemented with functional assessments in patients with neuropathic itch. The small unmyelinated C-fibers and thinly myelinated Aδ-fibers are of particular interest, as they transmit itch (41, 42). In quantitative sensory testing (QST), a validated test battery using thermal and mechanical standardized stimuli, detection and pain thresholds as well as response to suprathreshold stimuli are measured, allowing to infer a possible gain or loss of function of different nerve fiber populations (43–45). Additionally, QST informs about signs of neuronal sensitization, for instance by assessing mechanical allodynia and wind-up ratio (46). Although this non-invasive method provides a comprehensive neurophysiological profiling of sensory neuropathies, it is time-consuming, requires specialized personnel and the collaboration of the patient.

Large myelinated sensory fibers are not involved in itch transmission. Nevertheless, SFN may occur as part of a polyneuropathy with involvement of large fibers. Therefore, in patients with neuropathic pruritus due to a SFN, referral to a neurologist for nerve conduction studies or electromyography should be considered (3). Additionally patients with pruritic compression diseases may show pathological nerve conduction studies, as has been reported in brachioradial pruritus (47) and anogenital neuropathic itch (48).

Assessment of evoked potentials and microneurography constitute additional methods, in which functional impairment of selective nerve fibers are investigated (49, 50). These diagnostic procedures are mostly performed in research studies, but may be considered in selected clinical cases.

## Discussion

The diagnosis of neuropathic itch is challenging and may be overlooked in routine care. Neuropathic itch should be suspected in patients with chronic itch on normal appearing skin without a relevant systemic condition causing itch. A detailed medical history, especially in relation to the sensory symptoms, can further suggest the presence of a neuropathic origin. Typical is the presence of additional dysesthesias such as stinging and tingling, the occurrence of symptoms in attacks rather than continuously and alleviation with cool-packs or cold temperature. These clinical symptoms have been proposed as diagnostic criteria for pruritic SFN (5). However, these symptoms are not exclusive of neuropathic pruritic conditions. For instance, sensory symptoms other than itch such as crawling, tickling, and stinging have been reported in atopic dermatitis (51), while pain is perceived by the majority of patients with atopic dermatitis and psoriasis (52).

The localization of the dysesthesias, especially when distributed along a dermatome (e.g., in post-herpetic neuralgia or brachioradial pruritus) or in a *stocking-and-glove* distribution (e.g., in SFN), may yield further clues to the diagnosis of neuropathic itch and to the localization of the pathology within the somatosensory system (26).

Moreover, alloknesis, which can be easily tested by stimulating the affected skin area with a cotton wool or a brush, should be included in the evaluation of patients, as this phenomenon is characteristic of neuropathic itch, especially when neuronal sensitization has ensued (28).

Complementary exams are indicated to confirm a suspicious case of neuropathic itch. The determination of the IENFD, which is frequently reduced in neuropathic itch, is the gold-standard test to diagnose involvement of small-fibers. The biopsy needs to be performed at a pruritic non-lesional site, since the IENFD is also reduced in chronic scratch lesions (53) and in dermatoses (54–56), suggesting a neuropathic component of inflammatory pruritic conditions. However, while in chronic scratch lesions the IENFD normalizes with healing of the lesions, in neuropathic syndromes such as pruritic SFN and brachioradial pruritus the IENFD seems to be independent of the amount of scratch lesions present, arguing for an endogenous neuropathic mechanism leading to the rarefication of intraepidermal fibers (5, 35). A reduced IENFD is found not only in SFN, but also in extra-cutaneous neuropathic conditions such as brachioradial pruritus, likely due to anterograde transmission of neuromodulating factors leading to cutaneous neuroanatomical changes (57, 58).

Radiological exams, especially MRI, are useful when a neuropathic compression syndrome is suspected to identify orthopedic pathologies compromising neural structures. It should however be taken into account that in some cases anatomical abnormalities leading to neuropathic itch may be difficult to identify with medical imaging, as is the case in notalgia paresthetica (40).

In the management of patients with suspected neuropathic itch, diagnostic efforts focus on confirming the diagnosis and identifying the underlying pathological processes at the somatosensory system. With the advance of the understanding of the neurobiological mechanisms leading to neuropathic itch and their association with clinical signs and symptoms, a mechanism driven diagnostic work-up may be possible in the future, allowing the use of target specific drugs, which hopefully will result in a better care.

## Author Contributions

MP planned the manuscript, performed the literature search, wrote the manuscript, and approved the final version of the manuscript. HW performed the literature search, wrote the manuscript, and approved the final version of the manuscript. KA wrote the manuscript and approved the final version of the manuscript. SS planned the manuscript, wrote the manuscript, and approved the final version of the manuscript. All authors contributed to the article and approved the submitted version.

## Conflict of Interest

The authors declare that the research was conducted in the absence of any commercial or financial relationships that could be construed as a potential conflict of interest.

## Abbreviations

CT: computed tomography
DN4: *doleur neuropathique*
IENFD: intraepidermal nerve fiber density
IFSI: International Forum for the Study of Itch
QST: quantitative sensory testing
MRI: magnet resonance imaging
NP5: Neuropathic Pruritus 5
SFN: small-fiber neuropathy.
